# Indoor Radon Concentration and Risk Assessment in 27 Districts of a Public Healthcare Company in Naples, South Italy

**DOI:** 10.3390/life11030178

**Published:** 2021-02-24

**Authors:** Filomena Loffredo, Federica Savino, Roberto Amato, Alfredo Irollo, Francesco Gargiulo, Giuseppe Sabatino, Marcello Serra, Maria Quarto

**Affiliations:** 1 Advanced Biomedical Science Department, University of Naples, 80131 Naples, Italy; filomena.loffredo@unina.it (F.L.); serraemme@gmail.com (M.S.); 2 LB Business Services srl, 00135 Rome, Italy; savino.federica@gmail.com; 3 Occupational Health Service, Public Healthcare “Napoli 3”, 34102 Naples, Italy; r.amato@aslnapoli3sud.it; 4 Protection and Prevention Service, Public Healthcare “Napoli 3”, 34102 Naples, Italy; icratur@libero.it; 5 Building Division Office, University of Naples, 80138 Naples, Italy; francesco.gargiulo3@unina.it; 6 Advanced Metrological and Technological Services (CeSMA), University of Naples, 80138 Naples, Italy; giuseppe.sabatino@unina.it

**Keywords:** radon, effective dose, workplaces

## Abstract

Radon is a major source of ionizing radiation exposure for the general population. It is known that exposure to radon is a risk factor for the onset of lung cancer. In this study, the results of a radon survey conducted in all districts of a Public Healthcare in Italy, are reported. Measurements of indoor radon were performed using nuclear track detectors, CR-39. The entire survey was conducted according to a well-established quality assurance program. The annual effective dose and excess lifetime cancer risk were also calculated. Results show that the radon concentrations varied from 7 ± 1 Bq/m^3^ and 5148 ± 772 Bq/m^3^, with a geometric mean of 67 Bq/m^3^ and geometric standard deviation of 2.5. The annual effective dose to workers was found to be 1.6 mSv/y and comparable with the worldwide average. In Italy, following the transposition of the European Directive 59/2013, great attention was paid to the radon risk in workplaces. The interest of the workers of the monitored sites was very high and this, certainly contributed to the high return rate of the detectors after exposure and therefore, to the presence of few missing data. Although it was not possible to study the factors affecting radon concentrations, certainly the main advantage of this study is that it was the first in which an entire public health company was monitored in regards to all the premises on the underground and ground floor.

## 1. Introduction

Radon is a radioactive noble gas belonging to the ^238^U radioactive chain, produced by the decay of ^226^Ra [1]. It is ubiquitous in the earth’s crust in a concentration dependent on geology [2]. A fraction of radon produced in rocks and soils, escapes into the outdoor atmosphere where it is quickly diluted while, in confined spaces, it tends to accumulate reaching levels of concentration that are dangerous for health [3]. In 1988, radon was classified as a carcinogenic agent for humans by the International Agency for Research on Cancer (IARC) for evidence of an association between the exposure and onset of lung cancer [4]. Due to its long half-life (3.82 d), radon is almost completely exhaled after inhalation, otherwise, its progenies with short half-life, ^218^Po and ^214^Po, being electrically charged, can be attached to dust or smoke particles in indoor air. During the breathing process, they reach the bronchial tissue where they decay emitting radioactive alpha particles capable of damaging the pulmonary epithelium and thereby causing lung cancer. Radon and its progeny contribute more than 50% to the human exposure to natural ionizing radiation [5]. UNSCEAR [6] estimates that the annual effective dose for humans attributable to the radon exposure is about 1 mSv. After the residential exposure, the second important source of exposure to radon and its progenies, is the occupational exposure since people spend about 35% of their daytime in the workplace. Supported by the scientific evidence of epidemiological studies on residential exposure data [7,8,9,10,11,12] showing a statistically significant increase in the risk of lung cancer, due to the prolonged exposure to radon already at the level of 100 Bq/m^3^, the European Union has issued the Directive 59/2013 [13], which established a reference level for indoor radon concentrations in workplaces of 300 Bq/m^3^. Despite the European Union regulation, some authors [14,15,16] have questioned the correlation between the exposure to radon and the increased risk of lung cancer. They believe that the results obtained from the epidemiological studies are conditioned by the dose-response model adopted, the linear no-threshold (LNT). According to this model, the excess risk increases linearly versus the radon concentration but, these studies argue the lack of enough experimental data to support this thesis. Dobrzyński et al. [16] stated, in their meta-analysis, that there is no scientific evidence supporting the thesis that the exposure to radon is significantly correlated to the incidence of pulmonary cancer, at least for concentration values below 1000 Bq/m^3^.

In Italy, in July 2020, this Directive was transposed into the current National law [17] for the protection of health of the general population and workers against the ionizing radiation exposure. In addition, it imposes the action level for the home and workplaces of 300 Bq/m^3^. Despite this, in Italy, few surveys have been performed in the workplaces, many of them, on a local scale and with different methods of measurement. The aim of this study is to present the results of a radon survey conducted in 2018–2020 in the underground workplaces and on the ground floor at the Public Healthcare Company “Napoli 3” of Campania region, South Italy. Moreover, the annual dose estimation to workers and the excess lifetime cancer risk, were assessed using the data presented in the paper.

## 2. Materials and Methods

The survey conducted at the Public Healthcare Company “Napoli 3” was divided into four fundamental phases: (1) Sampling plan of the premises to be monitored; (2) exposure of the CR-39 detectors for two consecutive semesters in the sampled premises; (3) chemical etching and statistical analysis; (4) radon risk assessment. These steps are listed below.

### 2.1. Sampling Plan

The confined spaces to be monitored were selected by the Prevention and Protection Department of Public Healthcare Company “Napoli 3”. In this study, regardless of their intended use, all underground workplaces and on the ground floor, were monitored. The sampling plan concerned the 27 districts in which the Public Healthcare Company is divided and, a total of 607 rooms were sampled.

### 2.2. Monitoring, Detectors Analysis, and Statistical Analysis

The monitoring was conducted during 2018–2020 and involved a total of 1307 solid state nuclear detector CR-39 types. In each monitored workplace, two detectors were exposed for two consecutive semesters to obtain a whole year of exposure. Radon measurements were conducted according to the UNI ISO 11665-4: 2020 standard. CR-39 detectors were positioned about 2 m from the floor and at about 30 cm from the internal wall in order not to record the contribution from the Thoron. The quality assurance was performed by participating in the intercomparison exercise organized by the German Federal Office for Radiation Protection (BfS). Moreover, the laboratory of Radioactivity (lab.RAD, University of Naples Federico II, Naples, Italy) has a certification according to the ISO 9001:2015 standard and accreditation according to the European Standard EN ISO/IEC 17025 for the “integrated measurement method for determining average activity concentration of the radon 222 in the environment air using passive sampling and delayed analysis” (UNI ISO 11665:2020, part 4). 

After the exposure, all the CR-39 detectors were chemically etched using a solution of 6.25 M NaOH at (98 ± 1) °C for 60 min. The automatic counting of tracks density was performed by the Politrack system (mi.am s.r.l., Rivergaro, PC, Italy). It consists of an automated microscopic image analyzer equipped with a control computer and track analysis software. Finally, the radon concentration was calculated using Equation (1):

(1) $$C_{Rn}=\frac{N}{E\times T}$$

where N is the track density corrected by the background track density, E is the calibration factor, and T is the exposure time. The background track density was estimated to be 10 tracks/cm^2^ and it was determined by counting the tracks of a significant number of unexposed CR-39. The calibration factor was determined by exposing the detectors at a certified atmosphere in the range of exposure from 100 to 3000 Bq h m^−3^ at the National Metrological Institute (ENEA). The detection limit (LLD) of the method was estimated to be 4 Bq/m^3^ (with an exposure time of about 4320 h). To obtain the radon annual average concentration, the time-weighted average radon concentration from two consecutive 6-month periods, was calculated using the exposure time as weights, as shown in the following Equation (2):

(2) $$C_{Rn}^{T}=\frac{\left(\Delta t_{1}\;\times \;C_{Rn}^{1}\right)+\;\left(\Delta t_{2}\;\times \;C_{Rn}^{2}\right)\;}{\left(\Delta t_{1}+\;\Delta t_{2}\right)}$$

where Δ*t*_1_ and Δ*t*_2_ are the exposure times of two semesters and $C_{Rn}^{1}$ and $C_{Rn}^{2}$ are the integrated measured radon concentrations in the two semesters.

The central tendency of the radon measurements was described using the geometric mean since their distribution was skewed. The evaluation of normality of log-transformed data was tested by the Shapiro–Wilk test. All the statistical analysis was performed using the Stata software (Stata Corp, College Station, TX, USA).

### 2.3. Risk Assessment

The annual effective dose H to the workers due to the radon and its progeny was calculated as suggested by the Italian Legislation (Decreto Lagislativo 101/2020), using Equation (3) [14]:

(3) H(mSv/y)=C×T×D

where C is the radon concentration (Bq/m^3^), T is the occupancy factor, and D (6.7 × 10^−9^ Sv per Bq h m^−3^) is the dose conversion factor assuming an average equilibrium factor between radon and its daughters of 0.4 (ICRP 137) [18]. Generally, for a particular radionuclide, the internal dose is evaluated using two pieces of information, the intake and internal dose conversion coefficients called the dose per unit intake. Due to the short half-life of radon progenies responsible for the major contribution to the inhalation dose, it is not possible to perform bioassay measurements to assess the intake. Therefore, ICRP recommends the dose calculation using the activity concentration of radon in the atmosphere and the use of the dose conversion factor.

The excess lifetime cancer risk (ELCR) was estimated using the following Equation (4):

(4) ELCR=H×DL×RF

where H is the mean effective dose, DL is the average duration of life estimated to 70 years, and RF is the fatal cancer risk per Sievert (5.5 10^−2^ Sv^−1^) recommended by ICRP 103 [19].

The lung cancer cases per year per million persons (LCC) is estimated using the following Equation (5):

(5) LCC=H×RFLC

where RFLC is the risk factor lung cancer induction per million per person of 18 × 10^−6^ mSv^−1^ y reported in ICRP 50 [20].

## 3. Results

**Figure 1:**
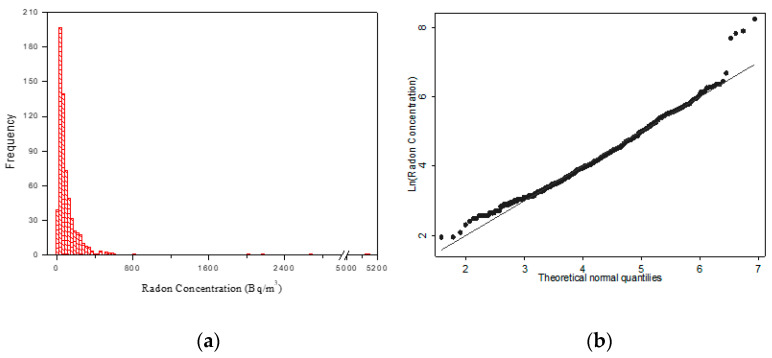
(**a**) Frequency distribution of indoor radon concentrations in workplaces of the Public Healthcare Company “Napoli 3”. (**b**) Q-norm plot of natural log-transformed radon concentration.

In this study, the results of a radon survey at the Public Healthcare Company “Napoli 3”, are reported. During the survey, 607 rooms were monitored using a total of about 1300 CR-39 detectors. After exposure, the return rate of the CR-39 detectors was 92% for the first semester and 90% for the second semester. In Figure 1a, the frequency distribution of the annual radon concentrations measured, is reported. Although the experimental data appear to show an approximately log-normal distribution, the Shapiro–Wilk test failed to assess normality (*p*-value < 0.001), as shown also in Figure 1b.

The minimum and maximum annual radon concentrations were found to be 7 ± 1 Bq/m^3^ and 5148 ± 772 Bq/m^3^, respectively, with a geometric mean of 67 Bq/m^3^, geometric standard deviation of 2.5, and the median value of 65 Bq/m^3^. Of all radon concentration measurements, 87% of the rooms presented radon concentrations lower than 200 Bq/m^3^, 8% had radon concentrations between 200 and 300 Bq/m^3^, and the other 5% had values greater than 300 Bq/m^3^.

**Figure 2:**
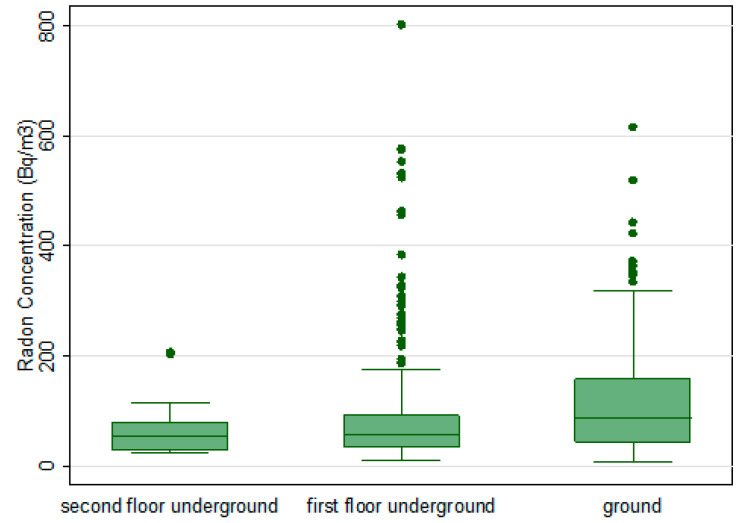
Relationship between radon concentrations and the floor of the monitored workplaces. Radon concentration values higher than 1000 Bq/m^3^ are not shown, in order to make the dependence of the average radon concentration appreciable on the floor where the measurements are performed.

The radon concentration varied with the floor where it was measured. In this survey, of all the monitored rooms, 43% were placed on the ground/underground floor, while 57% were placed on the first and second floor underground. The *t*-Student test on the log-transformed data presents a statistically significant dependence from the floor (*p* < 0.001; Figure 2). Here, it seems that the radon concentration increases with the floor, where the radon concentration in the underground is higher than those measured in the first and second floor underground. It is well known that the concentration of radon decreases as the floor increases, but we do not have an explanation to justify this different trend of radon concentration observed. In this study, no information on the characteristics of constructions, such as age, building material, and presence of forced ventilation affecting the radon concentration, that could explain this trend, were collected. However, our finding agrees with Ruano-Ravina et al. [21] that reported in their study an increase of radon concentration with height.

### Risk Assessment

Using Equation (3), the mean annual effective dose received by the workers was estimated to be 1.6 mSv/y, assuming an occupancy factor of 2000 h y^−1^ for a worker [22,23]. Moreover, assuming that the average duration of life is estimated to 70 years and the fatal cancer risk per Sievert is equal to 5.5 × 10^−2^ Sv^−1^ as recommended by ICRP 103, the mean excess lung cancer risk (ELCR) was found to be 0.62%. This estimate does not involve population-specific adjustments for major factors such as sex, age, and smoking habits. Finally, using the conversion factor for cancer cases per year per million per person of ICRP 50 [20] of 18 × 10^−6^ mSv^−1^ y, the lung cancer cases per million persons (LCC) was estimated to be 2.1 per million persons.

## 4. Discussion

In Italy, the culture of prevention to radon exposure in the workplace is still not widespread, although already in the year 2000, in accordance with the European Directive 96/29/EURATOM, the National Regulation was issued which regulated the protection of workers against the ionizing radiation exposure. Consequently, few surveys have been conducted and most of them have been performed on a local scale with different methods of measurement to determine the radon concentration in workplaces in some Italian regions [24,25,26,27,28,29]. The lack of mandatory monitoring in homes and workplaces not underground has meant that, although radon is a ubiquitous pollutant, the health risk that it can produce is little as considered by the general population, unlike other risks such as the toxicity of waste and the effects of electromagnetic fields. Studies [30,31] have shown that although people often have a high awareness of the presence of radon in their homes, they do not perceive a real risk and do not consider it necessary to monitor their living and working environments. On 31 July 2020, the Italian government has transposed the Council Directive 2013/59/Euratom into Decreto Legislativo 101 [17], which establishes the value of 300 Bq/m^3^ as the annual average of the radon concentration not to be exceeded in all closed environments, thus standardizing the value of the reference level to all work environments and homes. This study was conducted in an area with a high concentration of indoor radon and where building materials such as tuff and pozzolan, are often used. It is the first study conducted in Campania in which various types of workplaces were monitored, such as offices, hospitals, counseling centers, retirement homes for the elderly, regardless of the workers’ employment hours. The survey was conducted in the period in which the Italian government was enacting the law and the radon problem was beginning to be of great public interest. This is evidenced by the great participation that the workers of the monitored health company showed and by the high overall percentage of returned detectors during the survey of 97%.

The main results show that indoor radon concentrations were in general within the Italian legislation, in fact, about 95% of them are below 300 Bq/m^3^. The mean radon concentration of 118 Bq/m^3^ obtained in this study is higher than the national average of 75 Bq/m^3^ relative to the national survey carried out in homes [32]. Probably, this higher average value is attributable to the different lifestyle between homes and workplaces. In addition, the highest radon concentrations were found in some rooms that remained closed during the lockdown due to the COVID-19 health emergency. Our findings are in good agreement with previous studies carried out nationwide [33,34]. Moreover, these surveys conducted in different workplaces, spread throughout the Italian region, show an average radon concentration higher than obtained in national survey dwellings.

Numerous studies [1,35,36] report that indoor radon concentrations follow a lognormal distribution. However, our results show a departure from lognormality at a higher concentration level. The reason for this behavior could be attributed to the not too large sample size.

Moreover, the measurements were carried out in an area which was not homogeneous, either for the geology or for the characteristics of the buildings. This may justify the presence of outliers.

In the world, the estimate of the proportion of lung cancers attributable to radon ranges from 3 to 14% depending on the average radon concentration in the country concerned and the calculation methods implemented [37]. In the European pooling [9], the exposure-response relationship appeared to be approximately linear with no evidence for a threshold below which there was no risk. Conversely, Dobrzyński et al. [16], applying three different statistical models to analyze data from 34 radon studies, concluded that no statistical evidence could support the thesis that the linear model best fits the data over low radon concentrations.

In Italy, the total lung cancers attributable to radon is about 10% [38]. The risk of radon-induced lung cancer increases with exposure and the duration of the exposure, so it is very important to evaluate the annual effective dose to the workers. It is well known that the internal exposure to radon can be performed using either the epidemiological method or the dosimetric method. ICRP 126 [39], based on the re-assessment of dose coefficients considering the epidemiological studies on miners and the newest studies on residential exposures, concluded that the results of dose assessments using epidemiological data are similar to those using dosimetric models. In the present study, the assessment of the dose to workers was performed as recommended by the Italian law. This approach is based on the epidemiological method as recommended by ICRP 65. The annual effective dose to the workers due to radon indoor exposure was found to be 1.6 mSv/y. In Italy, national dose data relating to workplaces are not available. However, if we compare our data with exposures in homes, we observe that these values are higher than the Italian national average value which is 1.2 mSv/y [40]. Moreover, the mean annual effective dose is higher than the worldwide average of 1.15 mSv/y, reported by UNSCEAR [6]. The LCC found (2.1 per million persons) results lower than the limit range of 170–230 per million persons recommended by ICRP [22]. Our data show that the impact of radon exposure in these workplaces can be considered modest.

This study has some advantages, including being the first survey conducted in Campania in which all the rooms, at the underground and ground floor of a whole healthcare company, were monitored. Moreover, the radon concentrations measurements were conducted by applying a quality assurance system. The radon concentration data collected in this study could contribute to the radon map of Campania and to the validation of new analysis methods on the correlation between radon concentrations and geology [41,42].

The main limitation is represented by the fact that it was not possible to obtain information on the factors affecting indoor radon concentrations to correlate these to the major characteristics of the buildings, such as age of the construction and building materials.

## Data Availability

The data presented in this study are available on request from the corresponding author. The data are not publicly available due to the non-exclusive ownership by the authors. The data is shared with a third party, “Public Healthcare Company Napoli 3”.
